# Cerebrovascular Reactivity in Older Adults with Enlarged Perivascular Spaces and White Matter Hyperintensities

**DOI:** 10.1002/alz70862_109915

**Published:** 2025-12-23

**Authors:** Arunima Kapoor, Shubir Dutt, John Paul M Alitin, Allison C Engstrom, Trevor Lohman, Fatemah Shenasa, Aimée Gaubert, Amy Nguyen, Xingfeng Shao, Danny JJ Wang, Daniel A. Nation

**Affiliations:** ^1^ University of California, Irvine, Irvine, CA USA; ^2^ University of California San Francisco, San Francisco, CA USA; ^3^ Memory and Aging Center, UCSF Weill Institute for Neurosciences, University of California, San Francisco, San Francisco, CA USA; ^4^ Leonard Davis School of Gerontology, University of Southern California, Los Angeles, CA USA; ^5^ University of Southern California, Leonard Davis School of Gerontology, Los Angeles, CA USA; ^6^ University of Southern California, Los Angeles, CA USA; ^7^ Keck School of Medicine, University of Southern California, Los Angeles, CA USA

## Abstract

**Background:**

Enlarged perivascular spaces (ePVS) and white matter hyperintensities (WMH) are common and frequently co‐occurring features of cerebral small vessel disease. Few prior studies have examined the effect of cerebrovascular dysfunction on ePVS and WMH independently. In this study, we examined whether cerebrovascular reactivity (CVR)—an early and dynamic marker of cerebrovascular function—differs between individuals with no ePVS or WMH, only ePVS, only WMH and both ePVS and WMH to examine whether impaired cerebrovascular function confers appreciable risk to each of these pathologies.

**Method:**

Independently living older adults (*N* = 118, mean age = 68.4 years; SD = 6.9; age range 55‐89 years; 34.7% male, mean education = 16.4 years; SD = 2.4) free of dementia or clinical stroke were recruited from the community and underwent brain MRI. pCASL MRI quantified whole brain cerebral perfusion during vasodilation (CVR to hypercapnia) induced by visually guided breathing exercises (15s breath holds) and indexed by capnography. ePVS were scored using a 5‐point scale in the centrum semiovale (CS). WMH were scored using the Fazekas scale.

**Result:**

One‐way ANOVA revealed a significant difference in CVR between groups (no ePVS/WMH, ePVS, WMH, ePVS + WMH), *F*(3,114) = 3.37, *p* = .021, with medium effect size (partial η^2^ = .08). Post‐hoc comparisons indicated that the overall effect was driven by the difference between individuals with no ePVS or WMH (M = 9.89, SD = 3.65) and those with both (M = 6.57, SD = 2.40). Higher CVR was associated with decreased risk of ePVS + WMH, compared to all other groups, even after accounting for age, and sex (OR = 0.78, 95% CI (0.62, 0.98), *p* = .035).

**Conclusion:**

We explored the contribution of CVR to ePVS and WMH distinctly. These findings suggest that higher CVR substantially decreases risk of having combined WMH and ePVS‐CS. CVR deficits may represent early‐stage changes which contribute to the development of both ePVS‐CS and WMH. The cross‐sectional design of this study limits examination of directionality or bidirectionality. Future studies could explore how regional differences in CVR are associated with each pathology and whether that may differentially affect cognitive functioning in the long‐term.

